# MAKO robot-assisted total knee arthroplasty cannot reduce the aggravation of ankle varus incongruence after genu varus correction ≥ 10°: a radiographic assessment

**DOI:** 10.1186/s12891-023-06597-2

**Published:** 2023-06-15

**Authors:** Gang Jin, Yongyong Fan, Lingjun Jiang, Zhongyi Chen, Chenglong Wang

**Affiliations:** Department of Orthopaedics, Taizhou Hospital of Zhejiang Province affiliated to Wenzhou Medical University, Taizhou, 318000 Zhejiang P.R. China

**Keywords:** Ankle varus incongruence, Robot‑assisted, Total knee arthroplasty

## Abstract

**Introduction:**

The objective of this study was to investigate the ankle alignment alterations after the correction of knee varus deformity in MAKO robot-assisted total knee arthroplasty (MA-TKA).

**Methods:**

A retrospective analysis was conducted for 108 patients with TKA from February 2021 to February 2022. Patients were divided into two groups based on MAKO robot involvement during the procedure: the MA-TKA group (n = 36) and the conventional manual total knee arthroplasty (CM-TKA) group (n = 72). The patients were divided into four subgroups according to the degree of surgical correction of the knee varus deformity. Seven radiological measurements were evaluated pre and post-surgery: mechanical tibiofemoral angle (mTFA), mechanical lateral distal femoral angle (mLDFA), medial proximal tibial angle (MPTA), lateral distal tibial angle (LDTA), tibial plafond inclination angle (TPIA), talar inclination angle (TIA), and tibiotalar tilt angle (TTTA). TTTA is a quantitative representation of the extent of ankle incongruence.

**Results:**

The number of mTFA, mLDFA, and MPTA outliers in the MA-TKA group was significantly lower compared to the CM-TKA group (P<0.05). Knee varus deformity was properly corrected and the mechanical axis was restored in all patients, regardless of the treatment group. Only for varus corrections ≥ 10° did TTTA change significantly (p < 0.01) and ankle varus incongruence aggravate post-operation. The ΔTTTA correlated negatively with ΔTFA (r=-0.310,P = 0.001) and correlated positively with ΔTPIA (r = 0.490,P = 0.000). When the varus correction was ≥ 7.55°, the probability of ankle varus incongruence exacerbation increased 4.86-fold.

**Conclusion:**

Compared with CM-TKA, MA-TKA osteotomy showed more precision but was unable to reduce post-operation ankle varus incongruence. When the varus correction ≥ 10°, ankle varus incongruence aggravated, while when the varus correction ≥ 7.55°, the probability of ankle varus incongruence increased 4.86-fold. This may occasion the pathogenesis of ankle pain following TKA.

## Introduction

End-stage knee osteoarthritis (OA) is frequently accompanied by varus or valgus deformity. Total knee arthroplasty (TKA) is among the prevailing therapeutic options for end-stage OA [1–3], and the role of preparatory planning is critical for proper surgery [4]. As TKA’s degree of specialization improves, the survival rate of prostheses and patient satisfaction with TKA are increasing [5]. TKA effectively alleviates knee pain, improves range of motion, and permits recovery of the lower limb line of force by correcting knee varus or valgus [6].

Nonetheless, the change of mechanical axis can impact other joints of the lower extremities, thereby causing postoperative ankle pain [6–9]. Studies have shown that 24–35% of patients who undergo TKA develop concomitant ankle OA [10, 11]. The rising prevalence of ankle pain is garnering increased attention on post-TKA ankle malalignment. Correction of varus or valgus knee deformities may occasion ankle malalignment and increase the varus inclination of the ankle [12, 13]. Previous studies have demonstrated that ankle valgus incongruence aggravated following TKA [11]. However, the majority of ankle OA cases manifest ankle varus rather than valgus [14, 15]. Chang CB et al. [16] reported that in patients whose knee varus deformity was corrected ≥ 10 ° following TKA, the degree of ankle varus incongruence was aggravated. Shichman I et al. [17] revealed that A correction of ≥ 10 °in a genu valgum deformity can affect ankle joint alignment, leading to alterations in TPI and TI.

Robot-assisted total knee arthroplasty (RA-TKA), such as the Mako surgery robot (Stryker, USA), is gaining widespread utilization owing to advancements in TKA technology [18]. Numerous studies have indicated that compared to CM-TKA, RA-TKA can enhance accuracy, improve prosthesis position and limb force lines, and achieve superior imaging and functional results [18–21]. To the authors’ awareness, the ankle alignment alterations following MA-TKA is obscure, and there is no specific research on MA-TKA-induced modifications in ankle joint alignment. Consequently, the purpose of this study was to evaluate the post-MA-TKA alterations in ankle alignment after knee varus deformity correction.

## Materials and methods

A retrospective analysis was conducted for patients who underwent TKA surgery in our hospital from February 2021 to February 2022. Inclusion criteria comprised of: (1) patients with primary unilateral TKA due to end-stage varus knee OA (knee Kellgren-Lawrence (K-L) radiographic classification [22] of grade III or IV). Exclusion criteria consisted of: (1) patients diagnosed with infectious arthritis, traumatic arthritis, rheumatoid arthritis, or other autoimmune diseases; (2) the patient had neuromuscular dysfunction of lower limbs in the past; (3) severe medical and surgical diseases which prevent surgery; (4) patients with a previous lower extremity injury or history of surgery; (5) poor image quality and incomplete imaging data.

**Fig. 1:**
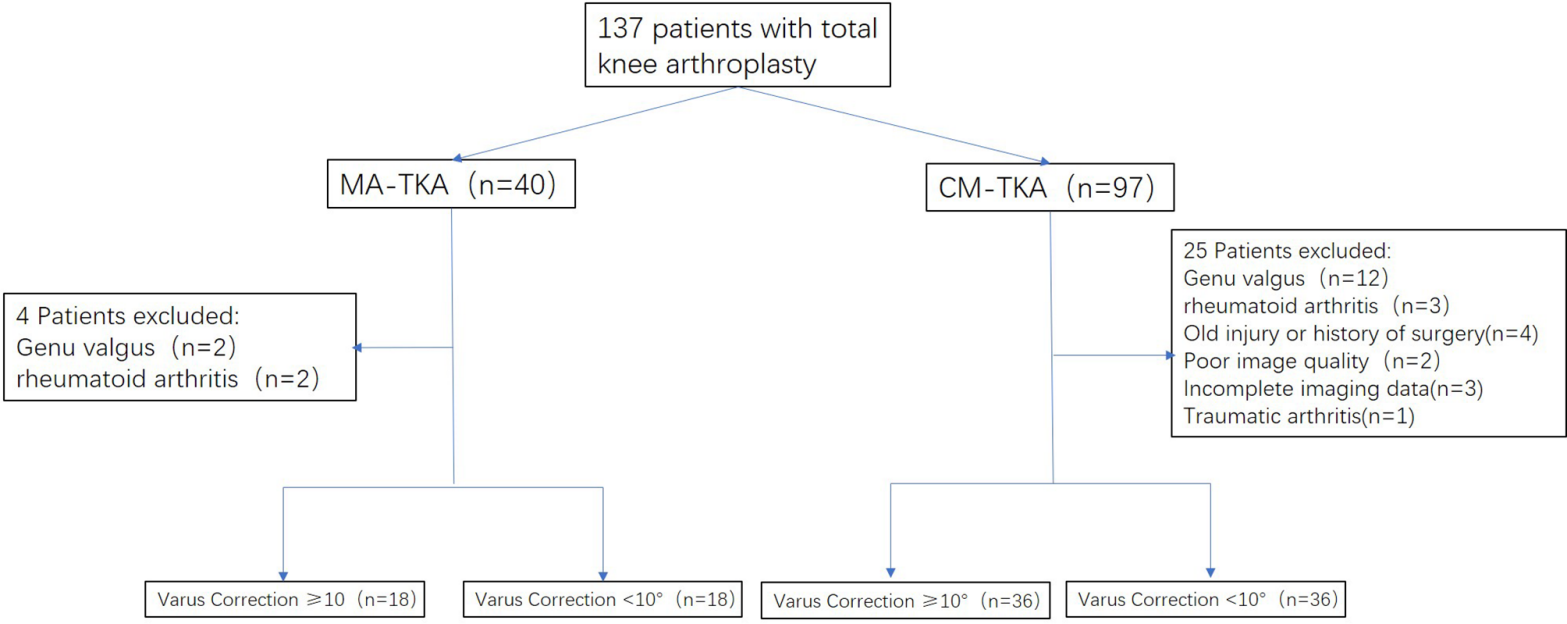
Flow chart for patient analysis

This study included 108 patients who were divided into two groups based on whether the operation was assisted by the Mako robot or not: the MA-TKA group (n = 36) and the CM-TKA group (n = 72). According to the degree of correction of the knee varus deformity during surgery, the patients were divided into four subgroups: group 1 (MA-TKA varus correction ≥ 10°), group 2 (MA-TKA varus correction ≤ 10°), group 3 (CM-TKA varus correction ≥ 10 °), and group 4 (CM-TKA varus correction ≤ 10°) (Fig. 1).

### Surgical method

All operations were performed by 2 surgeons with experience in TKA surgery. These surgeons have gone through their learning curve (intensive training and instruction in MA-TKA technology). In the MA-TKA group, CT scan of knee joint was performed before operation, and then 3D reconstruction model and preoperative plan were created by Mako total knee software. All patients were treated with Triathlon PS prosthesis and fixed platform (Stryker, USA). The TKA procedure was performed using a medial parapatellar approach and a tourniquet (from the beginning of the operation until the cement solidified and loosened). Femoral and tibial fixation pins and dynamic trackers were inserted at the upper edge of the patella and below the tibial tubercle during the operation. The intraoperative dynamic tracking, calculation of knee gaps, and coronal alignment via the MAKO system software. Surgeons recorded the planned bone resections after initial bone mapping, and when the recording was completed, the osteotomy began. When the green part of the osteotomy is completely cleared in the navigation view, it indicates that the current osteotomy has been completed. Further release of the joint capsule or ligament can be performed if necessary. After the osteotomy was completed, a trial mould of the prosthesis was installed and mobility and stability of the knee was assessed. Then install the prosthesis, suture the wound, and remove dynamic trackers and fixation pins of the femur and tibia.

In the CM-TKA group, the proximal tibia was osteotomized by extramedullary location, and then the distal femur was osteotomized by intramedullary localization.

### Radiographic measurement

Full-length weight-bearing X-ray films of lower extremities pre- and post-operation were secured for all patients, the images were digitized, and the data was analyzed using GE Healthcare Systems (Chicago, USA). Three orthopedic surgeons participated in the radiological measurement reliability test. A predetermined number of radiographic images were presented in random order by a research assistant who was not involved in the study. Subsequent to the reliability test, the surgeon measured all patients’ radiological indicators. The measurement method used in this study is based on reports by Chang CB et al. [10], Cooke TD et al. [23], and Moreland JR et al. [24].

**Fig. 2:**
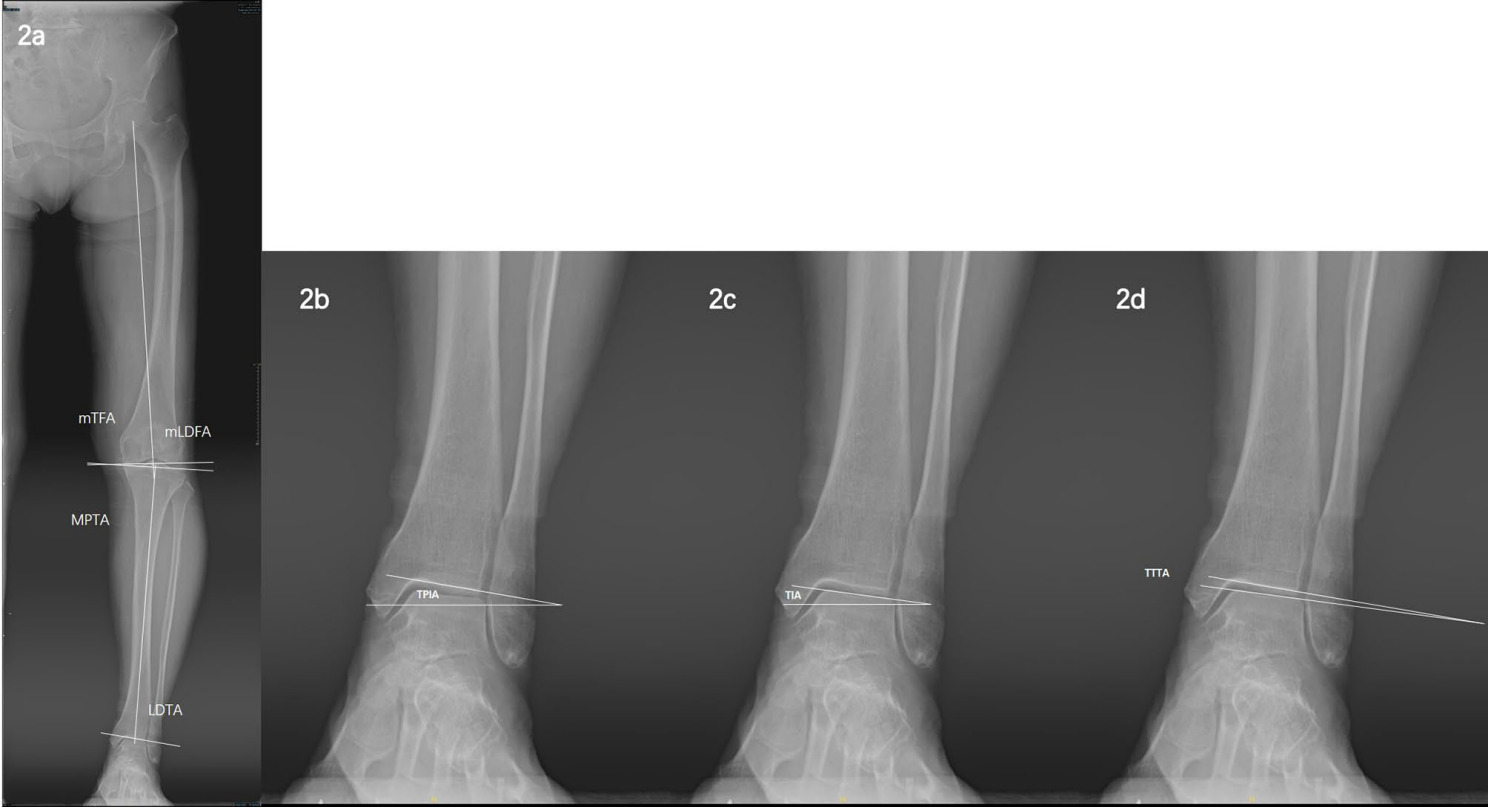
**(2a)** (1) The mechanical tibiofemoral angle (mTFA) is the angle between the mechanical axes of the femur and the tibia (optimally 180°). (2) The mechanical lateral distal femoral angle (mLDFA) is the angle between the femur’s mechanical axis and the femoral condyle’s tangent (optimally 90°). (3) The medial proximal tibial angle (MPTA) is the angle between the tibia’s mechanical axis and the tangent of the proximal tibia’s subchondral plate (optimally 90°). (4) The lateral distal tibial angle (LDTA) is the angle between the tibia’s mechanical axis and the tangent of the distal tibia’s subchondral plate (optimally 90°). **(2b)** The tibial plafond inclination angle (TPIA) is the angle between the tangent of the distal tibia’s subchondral plate and the horizontal line. **(2c)** The talar inclination angle (TIA) is the angle between the tangent of the talar dome’s subchondral plate and the horizontal line. **(2d)** The tibiotalar tilt angle (TTTA) is the angle between the tangent of the distal tibia’s subchondral plate and the talar dome’s tangent

The following radiographic measurements were made: (1) The mTFA is the angle between the mechanical axes of the femur and the tibia (optimally 180 °). (2) The mLDFA is the angle between the femur’s mechanical axis and the femoral condyle’s tangent (optimally 90 °). (3) The MPTA is the angle between the tibia’s mechanical axis and the tangent of the proximal tibia’s subchondral plate (optimally 90 °). (4) The LDTA is the angle between the tibia’s mechanical axis and the tangent of the distal tibia’s subchondral plate (ideally 90 °) (Fig. 2a). An outlier is defined as a difference ≥ 3 ° between the above-mentioned angle and the optimal value [25]. (5) The TPIA is the angle between the tangent of the distal tibia’s subchondral plate and the horizontal line (Fig. 2b). (6) The TIA is the angle between the tangent of the talar dome’s subchondral plate and the horizontal line. (Fig. 2c). (7) The tibiotalar tilt angle (TTTA) is the angle between the tangent of the distal tibia’s subchondral plate and the talar dome’s tangent (Fig. 2d). For TPIA, TIA, and TTTA, positive values represent the angular opening on the inside of the ankle, whereas negative values characterize the angular opening on the outside of the ankle. The differences between the postoperative and preoperative angles were expressed as ΔTPIA, ΔTTTA, and ΔTIA. As TTTA represents the consistency of the ankle angle, ankle varus incongruence can be described as the enlargement of the ankle’s lateral angle and Δ TTTA ≥ -1 ° [10].

### Statistical analysis

All data were analyzed using SPSS26.0 (IBM, Armonk, New York, USA). Under the assumption of single measurement and absolute agreement, the two-way random effect model was applied, and the intraclass correlation coefficient (ICC) was utilized to evaluate interobserver reliability. The sample size for the reliability test was calculated under the condition of an ICC target value of 0.8, with a 95% confidence interval width of 0.2. According to Bonnett’s approximation, the three raters’ interobserver reliability should be at least 36. The measurement data are described by mean ± standard deviation (SD), and the counting data are described by frequency and percentage. The Kolmogorov-Smirnov test was conducted to determine the data’s normality. The paired t-test was utilized to compare the variables prior and subsequent to the procedure. Two independent sample t-tests or Mann-Whitney U tests were performed to compare continuous variables between the two groups. The Chi-square test was applied to compare classified variables, while Pearson’s correlation coefficient was utilized to analyze the correlation between continuous variables. P < 0.05 was indicative of statistical significance.

## Results

### General information

In this study, 108 patients were enrolled. The MA-TKA group comprised 36 patients with an average age of 69.08 years (SD ± 7.49,50–83), of whom 9 were males (25%) and 27 were females (75%), and 19 cases involved right knees (52.8%) while 17 involved left knees (47.2%); the average BMI was 26.37 kg / m^2^ (SD ± 3.58). The CM-TKA group included 72 patients with an average age of 69.38 years (SD ± 6.22,58–85), of whom 26 were males (36.1%) and 46 were females (63.9%), and 42 cases involved right knees (58.3%) while 30 involved left knees (41.7%); the average BMI was 26.59 kg / m^2^ (SD ± 3.44). Demographic data did not differ between the two groups (Table 1). Regarding interobserver reliability, all radiographic measurements exhibited excellent ICC (Table 2).

**Table 1:** Demographic data for MA-TKA and CM-TKA groups

Parameter	MA-TKA(n = 36)	CM-TKA(n = 72)	P value
Age(y)	69.08 ± 7.49(50–83)	69.38 ± 6.22(58–85)	0.831
Gender			
Male	9(25%)	26(36.1%)	0.245
Female	27(75%)	46(63.9%)	
Operation side			
Right	19(52.8%)	42(58.3%)	0.583
Left	17(47.2%)	30(41.7%)	
BMI (kg/m^2^)	26.37 ± 3.58	26.59 ± 3.44	0.758
ASA			
2	32(88.9%)	62(86.1%)	0.685
3	4(11.1%)	10(13.9%)	
K-L grade			
III	10	18	0.756
IV	26	54	

**Table 2:** Interobserver reliability of radiographic measurements

ICC (95%IC)	mTFA	mLDFA	MPTA	LDTA	TPIA	TTTA	TIA
Preoperative	0.988 (0.979–0.993)	0.976 (0.959–0.987)	0.941 (0.900-0.967)	0.935 (0.885–0.965)	0.970 (0.949–0.984)	0.944 (0.906–0.969)	0.937 (0.892–0.966)
Postoperative	0.966 (0.942–0.982)	0.959 (0.929–0.977)	0.952 (0.887–0.978)	0.926 (0.876–0.959)	0.978 (0.961–0.989)	0.932 (0.885–0.962)	0.964 (0.938–0.981)

### Comparison of osteotomy accuracy between MA-TKA and CM-TKA groups

Prior to the operation, mTFA, mLDFA, MPTA, and LDTA did not differ significantly between the two groups (P > 0.05), but significant differences between the two groups were detected subsequent to the operation (P<0.05). The number of mTFA, mLDFA, and MPTA outliers in the MA-TKA group was significantly lower than in the CM-TKA group (P<0.05); however, there was no significant difference in LDTA (P > 0.05) (Table 3).

**Table 3:** Comparison of osteotomy accuracy between MA-TKA and CM-TKA groups

Parameter	MA-TKA(n = 36)	CM-TKA(n = 72)	P value
Preoperative			
mTFA(°)	171.53 ± 7.14	169.77 ± 5.72	0.168
mLDFA(°)	89.34 ± 2.48	88.54 ± 3.02	0.176
MPTA(°)	85.90 ± 1.98	85.52 ± 2.12	0.369
LDTA(°)	90.78 ± 3.53	90.13 ± 3.83	0.402
Postoperative			
mTFA(°)	180.98 ± 2.71	179.32 ± 3.56	0.015
Outlier(≥ 3°)	2(5.6%)	19(26.4%)	0.010
mLDFA(°)	90.13 ± 1.40	89.27 ± 2.57	0.026
Outlier(≥ 3°)	1(2.8%)	17(23.6%)	0.006
MPTA(°)	89.93 ± 1.38	89.19 ± 2.08	0.029
Outlier(≥ 3°)	1(2.8%)	14(19.4%)	0.018
LDTA(°)	90.78 ± 2.96	88.91 ± 3.27	0.005
Outlier(≥ 3°)	10(27.8%)	28(38.9%)	0.254

### Comparison of ankle angle alterations among different subgroups

In the group with MA-TKA varus correction ≥ 10 °, there were significant differences in mTFA, TPIA, TTTA, and TIA pre- and post-operation (P<0.01). In the group with MA-TKA varus correction < 10 °, there were significant differences in mTFA, TPIA, and TIA before and after surgery (P<0.05) but no significant difference in TTTA (P > 0.05). Interestingly, the CM-TKA group displayed similar results (Table 4).

**Table 4:** Comparison of ankle angle alterations among different subgroups

Parameter	Preoperative	Postoperative	P value
MA-TKA varus correction ≥ 10°(n = 18)			
mTFA(°)	167.59 ± 7.74	181.95 ± 3.05	0.000
TPIA(°)	5.26 ± 6.29	-2.80 ± 4.16	0.000
TTTA(°)	-0.23 ± 1.50	-1.52 ± 1.24	0.006
TIA(°)	5.49 ± 6.08	-1.28 ± 4.14	0.000
MA-TKA varus correction < 10°(n = 18)			
mTFA(°)	175.47 ± 3.48	180.02 ± 1.97	0.000
TPIA(°)	2.44 ± 5.38	0.27 ± 4.17	0.026
TTTA(°)	0.53 ± 2.34	0.96 ± 1.35	0.359
TIA(°)	1.91 ± 5.37	-0.68 ± 3.70	0.004
CM-TKA varus correction ≥ 10°(n = 36)			
mTFA(°)	166.26 ± 5.34	180.01 ± 4.18	0.000
TPIA(°)	6.00 ± 5.71	-3.91 ± 3.87	0.000
TTTA(°)	-0.58 ± 1.51	-1.99 ± 1.36	0.000
TIA(°)	6.58 ± 5.66	-1.92 ± 4.01	0.000
CM-TKA varus correction < 10°(n = 36)			
mTFA(°)	173.28 ± 3.55	178.63 ± 2.69	0.000
TPIA(°)	3.35 ± 5.37	-2.39 ± 4.14	0.000
TTTA(°)	0.00 ± 1.79	-0.13 ± 1.72	0.673
TIA(°)	3.35 ± 5.41	-2.26 ± 4.30	0.000

### Comparison of ankle angle between MA-TKA and CM-TKA following varus correction ≥ 10 °

There were no significant differences in TPIA, TTTA, and TIA between the two groups before and after the procedure (P > 0.05). No significant differences in ΔTPIA, ΔTTTA, and ΔTIA existed between the two groups (P > 0.05) (Table 5).

**Table 5:** Comparison of ankle angle between MA-TKA and CM-TKA following varus correction ≥ 10 °

Parameter	MA-TKA varus correction ≥ 10°(n = 18)	CM-TKA varus correction ≥ 10°(n = 36)	P value
Preoperative			
TPIA(°)	5.26 ± 6.29	6.00 ± 5.71	0.664
TTTA(°)	-0.23 ± 1.50	-0.58 ± 1.51	0.435
TIA(°)	5.49 ± 6.08	6.58 ± 5.66	0.520
Postoperative			
TPIA(°)	-2.80 ± 4.16	-3.91 ± 3.87	0.338
TTTA(°)	-1.52 ± 1.24	-1.99 ± 1.36	0.227
TIA(°)	-1.28 ± 4.14	-1.92 ± 4.01	0.587
Δ			
ΔTPIA(°)	-8.06 ± 4.40	-9.91 ± 3.99	0.127
ΔTTTA(°)	-1.29 ± 1.76	-1.41 ± 1.17	0.757
ΔTIA(°)	-6.77 ± 4.67	-8.49 ± 3.86	0.155

### Comparison of ankle angle alterations following varus correction ≥ 10° and < 10°

Before surgery, the mTFA in the correction ≥ 10 °group was smaller compared to the correction < 10 °group, and the TPIA and TIA were greater than in the correction < 10 °group (P<0.05); however, TTTA did not differ significantly between the two groups (P > 0.05). Following surgery, there were significant differences in mTFA, TPIA, and TTTA between the two groups (P<0.05), but no significant difference in TIA (P > 0.05). There were significant differences in ΔmTFA, ΔTPIA, ΔTTTA, and ΔTIA (p < 0.01) (Table 6).

**Table 6:** Comparison of ankle angle alterations following varus correction ≥ 10° and < 10°

Parameter	varus correction ≥ 10°(n = 54)	varus correction<10°(n = 54)	P value
Preoperative			
mTFA(°)	166.71 ± 6.20	174.01 ± 3.65	0.000
TPIA(°)	5.75 ± 5.86	3.04 ± 5.34	0.014
TTTA(°)	-0.461 ± 1.50	0.176 ± 1.98	0.063
TIA(°)	6.21 ± 5.77	2.87 ± 5.39	0.002
Postoperative			
mTFA(°)	180.65 ± 3.92	179.09 ± 2.54	0.016
TPIA(°)	-3.54 ± 3.96	-1.50 ± 4.30	0.012
TTTA(°)	-1.83 ± 1.33	0.24 ± 1.68	0.000
TIA(°)	-1.70 ± 4.02	-1.74 ± 4.14	0.968
Δ			
ΔmTFA(°)	13.95 ± 4.40	5.08 ± 2.29	0.000
ΔTPIA(°)	-9.29 ± 4.19	-4.55 ± 3.86	0.000
ΔTTTA(°)	-1.37 ± 1.38	0.06 ± 1.78	0.000
ΔTIA(°)	-7.92 ± 4.19	-4.60 ± 3.29	0.000

**Fig. 3:**
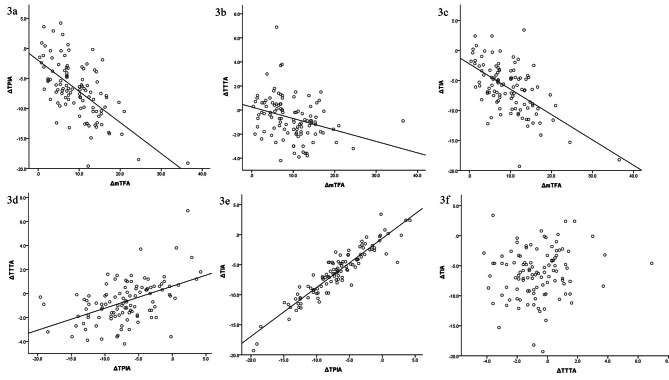
The dot chart depicts the correlations between the angles. ΔmTFA correlated negatively with ΔTPIA **(3a)**, ΔTTTA **(3b)**, and ΔTIA **(3c)**. ΔTPIA correlated positively with ΔTTTA (3d) and ΔTIA **(3e)**. There was no correlation between ΔTTTA and ΔTIA **(3f)**

ΔTTTA demonstrated a significant negative correlation with ΔmTFA (r = 0.310, P = 0.001) and a positive correlation with ΔTPIA (r = 0.490, P = 0.000), but no correlation with ΔTIA (r = 0.133, P = 0.085) (Fig. 3).

### The impact of correction degree on ankle varus incongruence

**Fig. 4:**
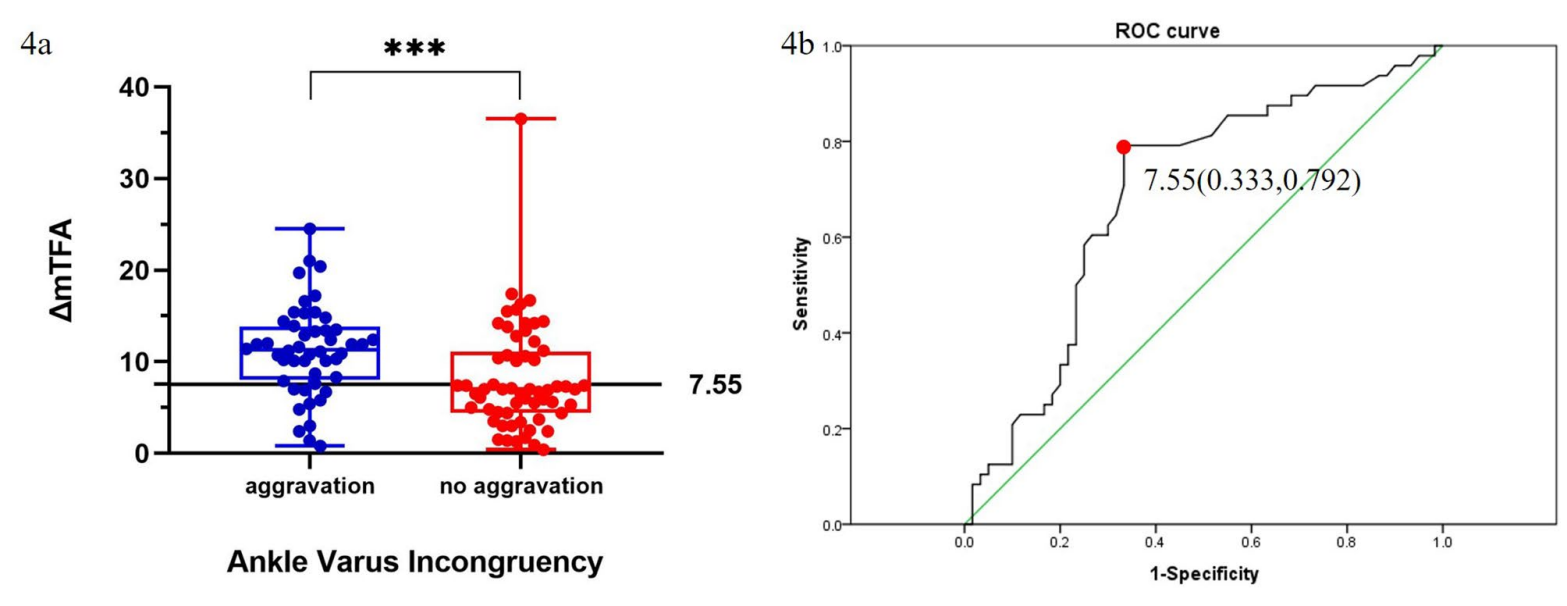
**(4a)** The box chart displays the distribution of patients with (aggravation) and without (no aggravation) ankle varus incongruence following TKA (x-coordinate), depending on the degree of Δ mTFA (y-coordinate). The cut-off value of the horizontal line was 7.55 °. **(4b)** Receiver operating characteristic curve. The cut-off value of ΔmTFA calculated by maximum 1-specificity and sensitivity was 7.55° [AUC 0.690 (0.588–0.793 95%CI), 1-specificity = 0.333, sensitivity = 0.792]. When the correction exceeded this critical value, the probability of ankle varus incongruence following TKA increased [OR = 4.86 (2.14–11.05 95% CI)]

The box chart indicates a significantly increased probability of ankle varus incongruence aggravation when ΔmTFA ≥ 7.55° (Fig. 4a). The ROC curve revealed that the ΔmTFA cut-off was 7.55 ° [AUC 0.690 (0.588–0.793 95%CI), 1-specificity = 0.333, sensitivity = 0.792] (Fig. 4b). When the correction exceeds this critical value, the probability of ankle varus incongruence following TKA increases [OR = 4.86 (2.14–11.05 95% CI)].

## Discussion

### Osteotomy accuracy was more precise in the MA-TKA group

In this study, MA-TKA was more accurate than CM-TKA for mTFA, mLDFA, and MPTA osteotomy (Table 3). Kayani B et al. [26] analyzed and compared the data of 60 patients with MA-TKA and demonstrated that compared with CM-TKA, MA-TKA enhanced the accuracy of implant location and limb alignment. Similarly, Batailler C et al. [18] revealed that MA-TKA induces less postoperative pain and improves prosthesis positioning compared with CM-TKA.

### The impact of different degrees of knee varus correction on ankle angle variations

Currently, the clinical correlation between ankle alterations and changes in knee alignment is not yet fully understood. Due to the lack of research on the effect of MA-TKA on the ankle force line, whether MA-TKA can reduce the ankle’s radiological index compared with CM-TKA was unknown. We analyzed and compared the ankle radiological changes in 36 MA-TKA cases and 72 CM-TKA cases after surgery. Our study demonstrated that TPIA, TTTA, and TIA postoperative alterations were statistically significant when knee varus correction ≥ 10° in both MA-TKA and CM-TKA groups (Table 4). When the correction was<10°, mTFA, TPIA, and TIA variations were statistically significant, but there was no difference in TTTA (Table 4). When the correction ≥ 10 °, Δ TTTA ≥-1 ° (Table 5); for a correction < 10 °, there was no difference in TTTA. Hence, we concluded that ankle malalignment was improved in both the MA-TKA and CM-TKA groups, and that ankle varus incongruence was only exacerbated when correction ≥ 10 °. This may be an underlying pathological mechanism of ankle pain following TKA.

Gao F et al. [12] demonstrated that CM-TKA rectifies knee varus or valgus deformities and ameliorates the ankle’s inclination; preoperative knee and ankle malalignment can be adjusted simultaneously following TKA. Kazemi SM et al. [27] reported that HTO significantly reduces the shear force on the ankle. Notably, studies by Tallroth K et al. [28] have indicated a putative association between knee and ankle OA and joint misalignment. Approximately 28.8% of knee OA patients have concurrent ankle OA. Lee JH et al. [11] revealed that OA appeared or progressed on X-ray films of the ankle in numerous cases following TKA. Ankle OA incidence rises with increasing talar tilt towards the ankle joint’s medial side before surgery or when the post-operation correction angle is substantial. Chang CB et al. [10] discovered that a considerable number of patients who underwent TKA had ankle OA and reduced hind foot flexibility. These patients’ ankle pain worsened following TKA, and the clinical results revealed exacerbations. Kim C et al. [29] demonstrated that residual varus deformity following TKA was associated with ankle pain. Modifications in knee alignment impact the alignment of the foot and ankle, thereby occasioning ankle symptoms. Nevertheless, the preoperative evaluation emphasizes the knee’s alignment, while pathological alterations of the ankle are frequently overlooked [30].

Shichman I et al. [17] reported that knee valgus correction ≥ 10 ° affects ankle alignment and induces modifications in TPIA and TIA, while knee varus correction ≥ 10 ° and < 10 ° had no effect on TPIA and TIA. They also found that TKA did not impact TTTA. However, Chang CB et al. [16] revealed that subsequent to knee varus correction ≥ 10 °, TPIA and TIA varus decreased, and the mechanical axis was restored while TTTA value increased, indicating an aggravation of ankle varus incongruence. This may facilitate ankle osteoarthritis progression [31] and exacerbate ankle pain. Our study demonstrated decreased TPIA and TIA post-operation in both the MA-TKA and CM-TKA groups (Table 4), signifying restoration of the patient’s mechanical axis following the procedure; this finding differs from that of Shichman I et al. [17]. However, knee varus correction ≥ 10 ° was associated with TTTA alterations (Table 4), corroborating Chang CB et al’s [16] report that ankle varus incongruence is aggravated. Subsequently, we compared and analyzed the effects of MA-TKA on ankle radiological indices. The results indicated that the Δ TPIA, Δ TTTA, and Δ TIA values in the MA-TKA group were numerically smaller than in the CM-TKA group when knee varus correction ≥ 10 °; however, there was no statistical difference (Table 5). Therefore, MAKO robot technology cannot prevent the aggravation of ankle varus incongruence following TKA.

### Comparison of ankle angle alterations in patients with varus correction ≥ 10 °and < 10 °group

Compared to the correction < 10 °group, the preoperative mTFA in the correction ≥ 10 °group was smaller, and the TPIA and TIA were greater, indicating more severe preoperative anomalies of the knee joint, distal tibia, and talus varus in the correction ≥ 10 °group. There was no difference in TTTA between the two groups; hence, ankle inclination did not differ significantly between the two groups prior to the operation. Postoperatively, lower limb malalignment was ameliorated to varying degrees. ΔTTTA in the correction ≥ 10 °group was greater than in the correction < 10 °group, with a value ≥-1 °; this is indicative of the exacerbation of ankle varus incongruence (Table 6).

Further analysis demonstrated a very significant correlation between ΔTTTA and ΔmTFA and ΔTPIA, but no correlation with ΔTIA (Fig. 3). It is inferred that TTTA modifications (which reflect the aggravation of ankle varus incongruence) are significantly related to mTFA and TPIA alterations and, by extension, to alterations in tibial alignment during TKA. In contrast to Chang CB et al’s findings [16], our results demonstrate that the negative value of ΔTTTA increases as ΔmTFA increases, indicating ankle varus incongruence deterioration (Fig. 3b). The box chart and ROC curve (Fig. 4) reveal that when the knee varus correction degree ≥ 7.55 °, the probability of ankle varus incongruence aggravation increased 4.86-fold.

There are some limitations to this study. Firstly, more data is still needed to make a strong statistical analysis of the results. Due to the lack of sufficient data on TKA patients with valgus deformity of the knee, this part can be further studied at a later stage. Secondly, this study predominantly analyzes the coronal arrangement, while the utilization of X-ray does not permit a precise evaluation of 3D objects. Thirdly, our data focus on the tibiotalar joint while excluding evaluations of the subtalar joint, which cannot be observed in the weight-bearing X-ray of the lower extremities. However, it can serve as a compensation mechanism for ankle alignment. Lastly, data analysis emphasized radiological evaluation while excluding ankle symptoms.

## Conclusion

In this study, we demonstrated that MA-TKA osteotomy had higher accuracy than CM-TKA. Despite MA-TKA’s ability to rectify knee varus deformity and restore the mechanical axis, it does not permit the reduction of postoperative ankle varus incongruence. When varus correction ≥ 10 °, ankle varus incongruence aggravated, while when varus correction ≥ 7.55 °, the probability of ankle varus incongruence increased 4.86-fold, which may be the underlying pathogenesis of ankle pain following TKA.

## Data Availability

The data that support the findings of this study are available from Taizhou Hospital of Zhejiang Province but restrictions apply to the availability of these data, which were used under license for the current study, and so are not publicly available. However, data can be obtained from the corresponding author with reasonable request and permission from Taizhou Hospital in Zhejiang Province.
